# Idiopathic erythrocytosis: a germline disease?

**DOI:** 10.1007/s10238-023-01283-y

**Published:** 2024-01-20

**Authors:** E. M. Elli, M. Mauri, D. D’Aliberti, I. Crespiatico, D. Fontana, S. Redaelli, S. Pelucchi, S. Spinelli, B. Manghisi, F. Cavalca, A. Aroldi, A. Ripamonti, S. Ferrari, S. Palamini, F. Mottadelli, L. Massimino, D. Ramazzotti, G. Cazzaniga, A. Piperno, C. Gambacorti-Passerini, R. Piazza

**Affiliations:** 1 Division of Hematology and Bone Marrow Transplant Unit, Fondazione IRCCS, San Gerardo dei Tintori, Monza, Italy; 2 Department of Medicine and Surgery, University of Milano - Bicocca, Monza, Italy; 3 Tettamanti Research Center, IRCCS, San Gerardo dei Tintori, Monza, Italy; 4 Monza and Brianza Foundation for the Child and his Mother (MBBM), IRCCS, San Gerardo dei Tintori, Monza, Italy

**Keywords:** Myeloid Neoplasia, Idiopathic erythrocytosis, Erythropoiesis, NGS sequencing

## Abstract

Polycythemia Vera (PV) is typically caused by V617F or exon 12 *JAK2* mutations. Little is known about Polycythemia cases where no *JAK2* variants can be detected, and no other causes identified. This condition is defined as idiopathic erythrocytosis (IE). We evaluated clinical-laboratory parameters of a cohort of 56 IE patients and we determined their molecular profile at diagnosis with paired blood/buccal-DNA exome-sequencing coupled with a high-depth targeted OncoPanel to identify a possible underling germline or somatic cause. We demonstrated that most of our cohort (40/56: 71.4%) showed no evidence of clonal hematopoiesis*,* suggesting that IE is, in large part, a germline disorder. We identified 20 low mutation burden somatic variants (Variant allelic fraction, VAF, < 10%) in only 14 (25%) patients, principally involving *DNMT3A* and *TET2*. Only 2 patients presented high mutation burden somatic variants, involving *DNMT3A*, *TET2*, *ASXL1* and *WT1*. We identified recurrent germline variants in 42 (75%) patients occurring mainly in *JAK/STAT*, *Hypoxia* and *Iron metabolism* pathways, among them: JAK3-V722I and HIF1A-P582S; a high fraction of patients (48.2%) resulted also mutated in homeostatic iron regulatory gene HFE-H63D or C282Y. By generating cellular models, we showed that JAK3-V722I causes activation of the JAK-STAT5 axis and upregulation of EPAS1/HIF2A, while HIF1A-P582S causes suppression of hepcidin mRNA synthesis, suggesting a major role for these variants in the onset of IE.

**Graphical abstract:**

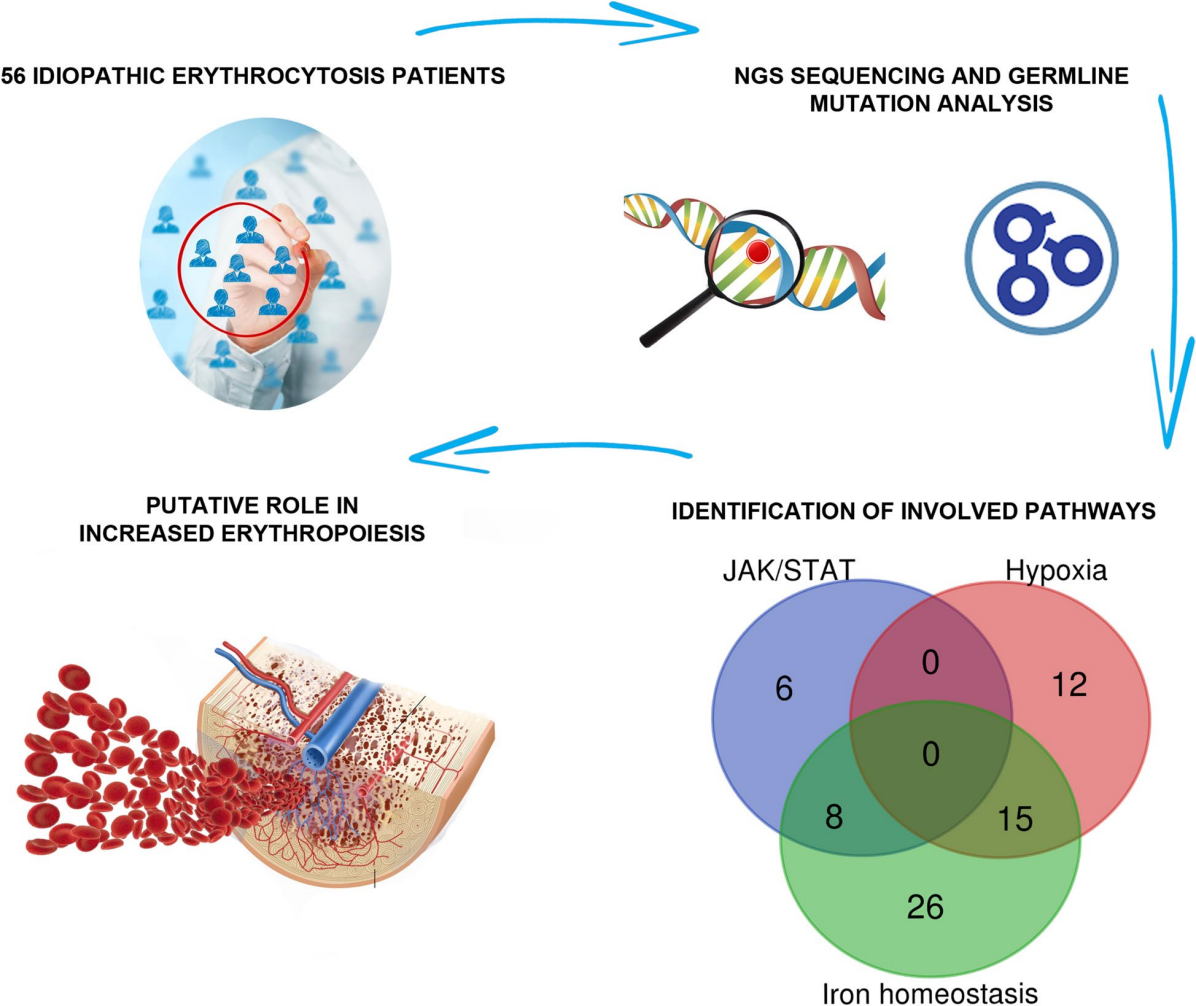

**Supplementary Information:**

The online version contains supplementary material available at 10.1007/s10238-023-01283-y.

## Introduction

The term “erythrocytosis” or “polycythemia” refers to an absolute or relative increase in hemoglobin (Hb)/hematocrit (Hct) levels from baseline sex-, race-, and altitude-adjusted normal values 1. In 2016, the World Health Organization (WHO) lowered the proposed Hb and Hct diagnostic thresholds for Polycythemia Vera (PV) to 16.5 g/dL/49% and 16 g/dL/48% for men and women, respectively 2. As these reference values were generated in large part using Caucasian people as reference, other criteria were added to facilitate diagnosis, including: (1) Hb o Hct level greater than 99th percentile of reference range for age, sex, or altitude of residence; (2) a red cells mass (RCM) that is at least 25% above mean normal predicted value 3.

Erythrocytosis can have a primary or secondary origin [4–6, as summarized in Table 1.

**Table 1:** Causes of erythrocytosis

Primary erythrocytosis
Congenital: EPO receptor mutations Acquired: Polycythemia vera
Secondary erythrocytosis
Congenital Oxygen-sensing pathway mutations: * VHL* gene mutations (Chuvash erythrocytosis) * EGLN1* gene mutations * HIF1A/EPAS1* gene mutations * EPO* gene mutations High oxygen-affinity hemoglobin Methemoglobinemia Bisphosphoglycerate mutase deficiency
Acquired (a) EPO mediated: Oxygen-sensing pathway mutations Central hypoxic process (Chronic lung disease, Right to left cardiopulmonary vascular shunts, Carbon monoxide poisoning, Smoker’s erythrocytosis, hypoventilation syndromes, High-altitude) EPO mediated Eisenmenger syndrome Local renal hypoxia (renal artery stenosis, End-stage renal disease, renal cysts (polycystic kidney disease), Post-renal transplant erythrocytosis (b) Pathologic EPO production: Tumors (Cerebellar hemangioblastoma, Meningioma, Parathyroid carcinoma/adenomas, Hepatocellular carcinoma, renal cell cancer, Pheochromocytoma, Uterine leiomyomas) Drug associated (Erythropoietin administration, Androgen administration) Mineral toxicity (cobalt, nickel, manganese)
Idiopathic erythrocytosis

Primary Erythrocytosis identifies various conditions characterized by an intrinsic defect of the erythroid progenitor cells responsible for an increase in the RCM and Hb levels. The most common cause of acquired primary erythrocytosis is the PV, a clonal myeloproliferative neoplasm caused by somatic, activating mutations occurring principally on the *JAK2* tyrosine kinase. In contrast, a secondary erythrocytosis arises when the increased red cell production is driven by factors external to the erythroid compartment, such as increased erythropoietin (EPO) production for any reason. Primary and secondary causes can be classified further as either congenital or acquired [4–6.

Once all the causes of primary and secondary erythrocytosis have been considered, there remains a group for which no cause can be identified. This condition is known as idiopathic erythrocytosis (IE). The frequency of IE has been estimated to be 1.1 per 1000 subjects, which is higher than that observed in PV 7. Heterogeneous mechanisms underlying IE have been suggested, including ‘early’ PV and unrecognized secondary or congenital polycythemia. However, the transition of a patient initially classified as IE into PV is a rare occurrence 7. IE shows a trend for a stable disease with low thrombotic risk and low tendency to spontaneous progression to myelofibrosis. However, thromboembolic events have been described in young patients 8. Evidence is lacking to define best management, but aspirin and venesection to a target Hct should be considered. Cytoreductive therapy, to date, is not appropriate for the treatment of patients with erythrocytosis in whom there is no evidence of a malignant clone 9, 10.

To gain insight into the molecular mechanisms responsible for the onset of IE, here we analyzed clinical-laboratory features and molecular profile of 56 IE patients, to identify a possible underling germline or somatic variant responsible for the onset of IE.

## Patients and methods

### Patients and study design

**Fig. 1:**
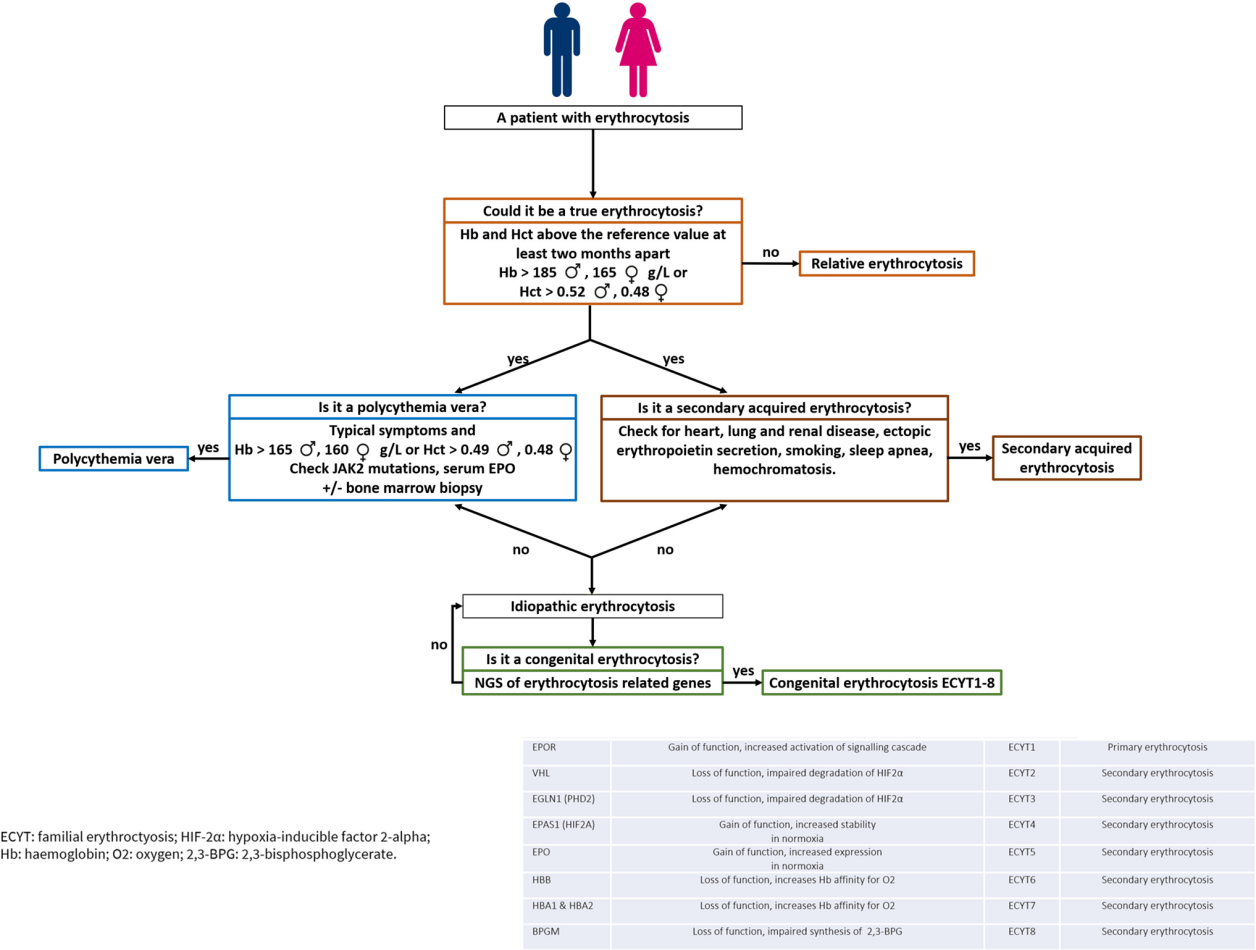
Simple three-step clinical algorithm for the diagnosis of erythrocytosis (from *Diagnosis and management of non-clonal erythrocytosis remains challenging: a single center clinical experience*; Doma et al. Annals of Hematology, 2021; modified) Abbreviations: Hb: hemoglobin; Hct: hematocrit, NGS: next-generation sequencing; ECYT: familiar erythrocytosis; HIF-2ɑ: Hypoxia-inducible factors 2-alpha; O_2_: oxygen; 2–3-BPG: 2.3-bisphosphoglycerate; EPO: serum erythropoietin

At the Hematology Division of San Gerardo Hospital, between 1999 and 2021, we identified a cohort of 56 Caucasian adult IE patients by a routine three-step diagnostic work-up 7 summarized in Fig. 1.

The diagnosis of PV was evaluated according to the 2016 WHO diagnostic criteria 2; all the known causes of secondary erythrocytosis, familiar or acquired as well as other form of primary and secondary erythrocytosis were excluded by routinary laboratory and radiological tests 11, 12.

IE patients were identified after exclusion of congenital causes according to patient’s history and next-generation sequencing (NGS), performed with peripheral blood (PB) samples collected at diagnosis, for genes linked to congenital erythrocytosis 5.

By using an electronic case report form (e-CRF), we collected clinical and demographic relevant parameters, including complete blood count, EPO and ferritin levels, histological bone marrow (BM) specimen and thrombosis at presentation and during follow-up as well as data concerning progression into the overt myelofibrosis, leukemic transformation, secondary neoplasia and death.

In particular BM biopsies were performed at diagnosis, where necessary to exclude PV, as indicated by 2016 WHO criteria 2 and during the follow-up, when the clinical picture was suggestive for MF or leukemia evolution. BM specimen was not performed at onset only in those rare cases with sustained absolute extreme erythrocytosis, represented by Hb levels > 18.5 g/dL in men (Hct, 55.5%) or 16.5 g/dL in women (Hct, 49.5%), or in presence of non-canonical *JAK2* mutations or clonal somatic myeloid mutations with VAF > 2%.

All IE patients were treated with phlebotomies, in order to maintain Hct < 50% and, when appropriate, they received low dose aspirin, according to our internal, evidence-based protocol for the management of venesection, which is shared among all principal Italian hematology and transfusion centers 8, 10. These patients received cytoreductive treatment (principally hydroxyurea) if they presented thrombotic complication and/or relevant need of phlebotomies. All patients were followed until death or data cut-off.

As clinical controls, we enrolled 56 consecutive PV patients, strictly diagnosed in agreement to 2016 WHO criteria 1, 8. All PV patients were treated with phlebotomies, in order to maintain Hct < 45%, associated with low dose aspirin and cytoreduction based on cardiovascular risk stratification, according to current guidelines 13.

Paired blood/buccal-DNA exome-sequencing as well as NGS myeloid panel were performed to identify somatic or germline mutations responsible for the onset of the disease.

### Ethics

IE patients provided written informed consent, which was approved by the institutional ethics committee. This study was performed in accordance with the standards of the Helsinki Declaration. The promoter of this study is the Department of Medicine and Surgery of the University of Milano—Bicocca, IRCCS, San Gerardo dei Tintori, Monza. The study (protocol 212) was approved by the Ethics Committee of ASST Monza on 14 December 2015 (Title: *Identification and characterization of the somatic lesions responsible for the onset of Acquired Idiopathic Erythrocytosis*—Code assigned: CE 0069761/15).

### Molecular analyses

The JAK2-V617F mutation was detected by allele-specific PCR according to the protocol of Baxter et al. 14*JAK2* exon 12 variants were determined by Sanger sequencing 15.

NGS analysis was performed using DNA extracted from PB by the Myeloid Panel (Sophia Genetics™), a commercially available NGS panel of current use in specialized laboratory for clinical practice, which allows sequencing of 30 genes mostly involved in myeloid malignancies 16. The minimal coverage was 1000x.

For a subset of 27 IE patient genomic DNA from BP buffy coat (*bona fide* tumor DNA) and buccal mucosa (germline DNA) was extracted and exome-sequenced. Capture was performed with SureSelect Human All Exon V6 kit (Agilent). Mean exonic coverage was > 120x, with an on-target capture between 50 and 60%.

### Data availability

Raw sequencing data support the findings of this study are openly available in the SRA repository at the following link: https://www.ncbi.nlm.nih.gov/sra/PRJNA965921.

### Bioinformatics

Primary analysis and variant-calling were done as previously described 17, 18. Given the absence of a clear underlying inheritance pattern we imposed a very mild pre-filtering strategy, keeping all the variants not associated with a specific polymorphism and those showing a MAF < 0.2. Hypergeometric tests were then used to test the enrichment of specific ontologies (FDR-adjusted padj < 0.1). SORVA scripts were generated for candidate target genes.

### Plasmids and transfections

Stable K562S cell lines were retrovirally infected using 293 FT packaging cells transfected with 10 μg of 217EX-U0982-Lv224/225 vector (Genecopoea) encoding wild-type or mutated *JAK3, EPAS1* or *HIF1A* cDNA variants. Transfection was performed using the JetPrime transfection reagent (PolyPlus). Retroviruses were collected after 3 days of culture. Primers for site-directed mutagenesis are reported in Table S1.

Details about molecular procedures and bioinformatics methods were described in Supplementary information.

## Results

The principal clinical and laboratory features of IE at diagnosis are reported in Table 2, together with a PV control group. A complete database of clinical features and mutations identified is available in the supplementary information section. IE patients were more frequently male (Table 2: 82%, *p* = 0.002) and younger than PV control groups (median age 53.6 *vs* 62.5 years, in IE *vs* PV patients, respectively, *p* = 0.001). At onset of disease, platelets count was significantly higher in PV than IE (*p* = 0.001); while, Hb levels were comparable in both disorders (*p* < 0.05). Median White Blood Cell (WBC) count was lower in IE than PV (*p* = 0.004) but within normal range in both diseases. A trend toward a higher age at diagnosis for female IE patients was also noted (median age 60 *vs* 67.5 years in male *vs* women, respectively; *p* = 0.20). The presence of palpable splenomegaly was rare in IE while common in PV patients (3.6% *vs* 31% in IE *vs* PV, respectively; *p* = 0.0002). EPO plasma levels in IE cohort were in the normal range and consistently higher than in PV patients (8.8 *vs* 1.9 IU/L, *p* = 0.0001). Interestingly, ferritin levels were also higher in IE cohort than in PV cases (115 *vs* 23 ng/mL; *p* = 0.0003), suggesting a more efficient iron uptake in the IE group.

**Table 2:** Clinical features of Idiopathic Erythrocytosis (IE) and Polycythemia Vera (PV) patients

Clinical feature	*N* = 56 pts (IE)	*N* = 56 pts (PV)	*p* value
Male/female, *n* (%)	46/10 (82.1/17.9)	31/25 (55.4/44.6)	0.002
Age (years), median (range)	53.6 (16.2–77.3)	62.5 (24.9–78.6)	0.0001
Hb (g/dl), median (range)	18 (16.2–23.1)	18.1 (15.7–22.2)	ns
Hematocrit (%), median (range)	52.3 (47–67.6)	54.9 (48–70.2)	ns
WBC count (× 10^9^/L), median (range)	7.3 (4.3–15.6)	9.5 (4.7–19)	0.004
PLT count (× l0^9^/L), median (range)	202 (100–347)	437 (139–841)	0.001
Serum erythropoietin (U/L), median (range)	8.8 (1–25.5)	1.9 (0.6–294)	0.0001
Ferritin level (ng/mL), median (range)	115 (2–363)	23 (4–346)	0.0003
Palpable splenomegaly, *n* (%)	2(3.6)	17 (30.3)	0.0002
Previous thrombosis, *n* (%)	9 (16)	14 (25)	ns
Post thrombosis, n (%)	6 (10.7)	6 (10.7)	ns
Antiplatelet therapy, *n* (%)	42 (75)	55 (98.2)	0.0003
MF evolution, *n* (%)	2(3.6)	10 (17.8)	0.01
AML evolution, *n* (%)	0 (0)	2 (3.6)	nv
Deaths, *n* (%)	3 (5.3)	5 (8.9)	ns

*Hb* hemoglobin, *MF* myelofibrosis, *AML* acute myeloid leukemia

*nv* not evaluable

*ns* not significant

Most of IE patients (40/56: 71.4%) showed no evidence of clonal hematopoiesis, supporting the hypothesis that IE are in large part caused by a non-neoplastic disorder*.*

**Fig. 2:**
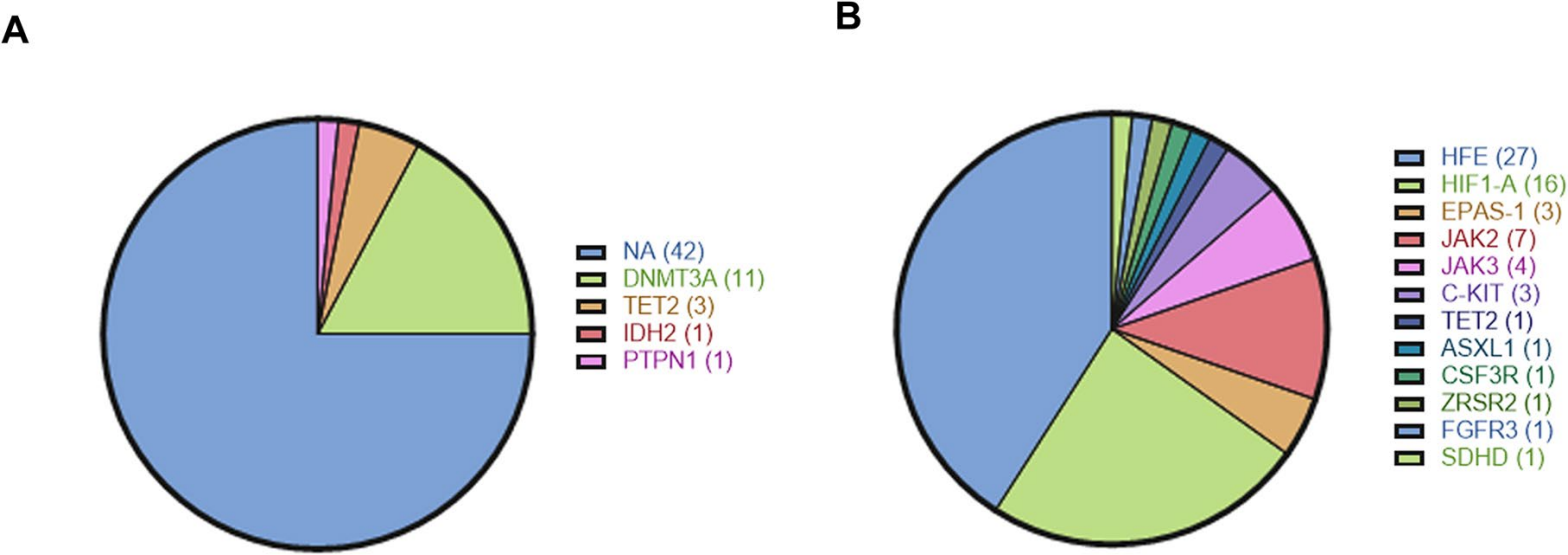
**A** Distribution and molecular profile of somatic variants in IE patients; **B** Distribution and molecular profile of germline variants in IE patients

In 14 IE patients (25%), we identified 16 Low Mutation Burden somatic variants (variant allele frequency—VAF < 10%), principally involving *DNMT3A* (19.6%) and *TET2 (*5.4%) genes (Fig. 2A and Table S2A). In only 2 cases, we found evidence of high VAF somatic variants occurring in *DNMT3A*, *TET2*, *ASXL1* and *WT1* genes.

This suggests that a large fraction of IE may be caused by a germline disorder, functionally connected with congenital erythrocytosis but characterized by adult onset and limited penetrance 19.

**Fig. 3:**
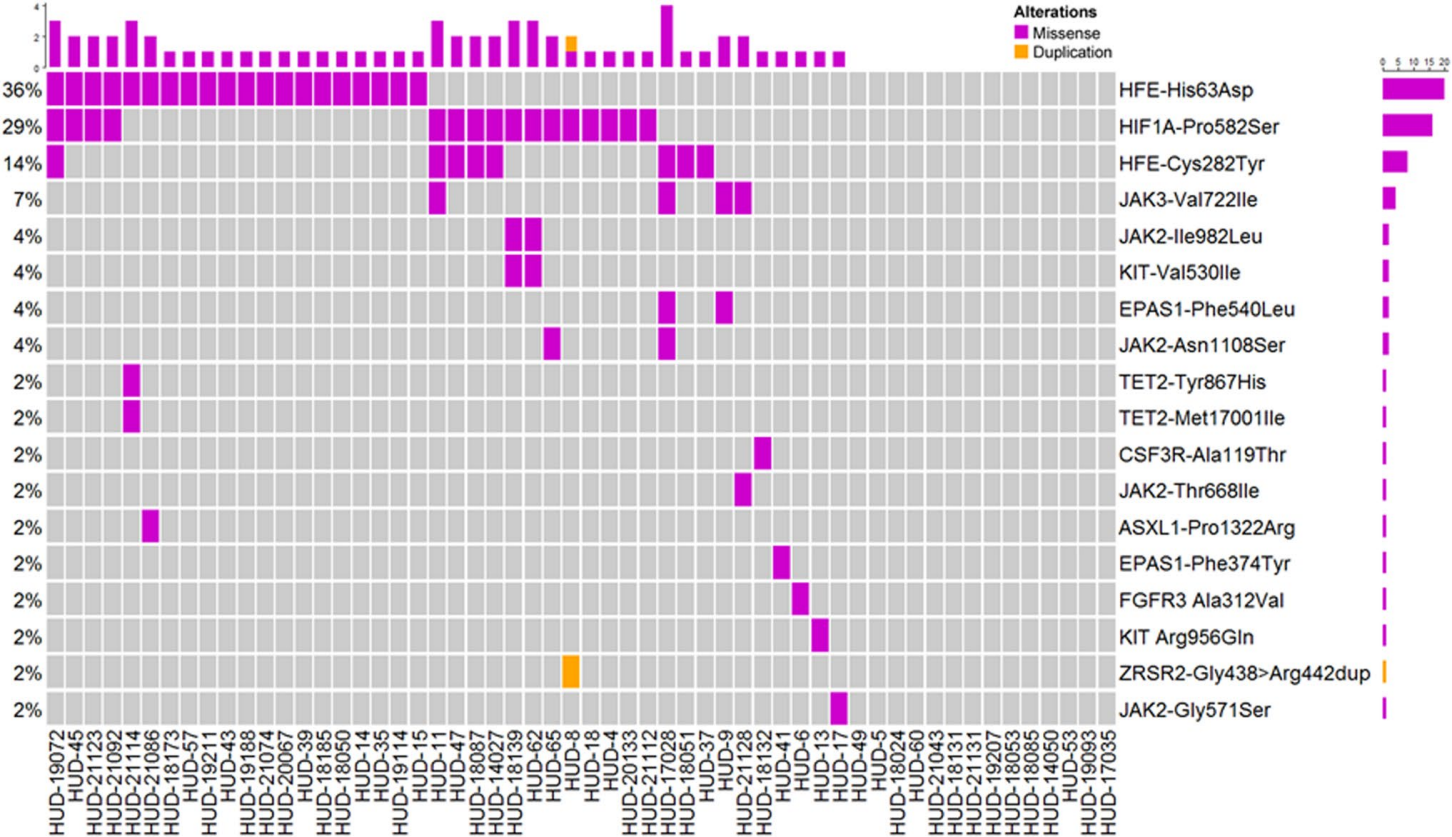
OncoPrint plot reporting the germline variants associated with the JAK-STAT pathway, Response to hypoxia, and Cellular iron ion homeostasis Gene Ontologies enriched in the IE patients. Purple tiles indicate missense mutations, orange tiles duplications and grey ones absence of mutation

To isolate the possible candidate pathogenic variants in the group of IE patients without signs of clonal hematopoiesis, we focused on polymorphisms occurring on genes associated with the JAK-STAT pathway, *Response to hypoxia*, and *Cellular iron ion homeostasis* gene ontologies (Tables S3, S4 and S5). We applied the SORVA framework to this gene subset 20 to assess the statistical significance of individual variants potentially associated to the IE phenotype. This approach allowed us to identify a set of variants significantly enriched in the IE phenotype, namely: JAK2 p.N1108S, JAK2 p.G571S, JAK2 p.I982L, JAK3 p.V722I, HFE (homeostatic iron regulatory) p.C282Y, HFE p.H63D, HIF1A p.P582S, EPAS1 p.P540L and EPAS1 p.F374Y (Figs. 2B, 3 and Table S2B).

Notably, the high fraction of *HFE*-mutated patients (27/56, 48.2%; Table S2B) and the high prevalence of males in our study group (82.1%; Table 2) are in line with a report showing comparable frequencies of HFE-mutated cases in a different IE cohort 21, therefore supporting the idea that mutated *HFE* is functional for the onset of IE and not a spurious finding.

Overall, we identified recurrent germline variants occurring in JAK/STAT, *Hypoxia* and *Iron metabolism* pathways in 42 (75%) patients, with a median of 2 variants/patient (Fig. 3).

To investigate the functional role of these variants, we generated cellular models for JAK3 p.V722I, EPAS1 p.P540L, EPAS1 p.F374Y and HIF1A p.P582S, since the effect of these variants at molecular level is still largely unknown.

### JAK3-p.V722I model

**Fig. 4:**
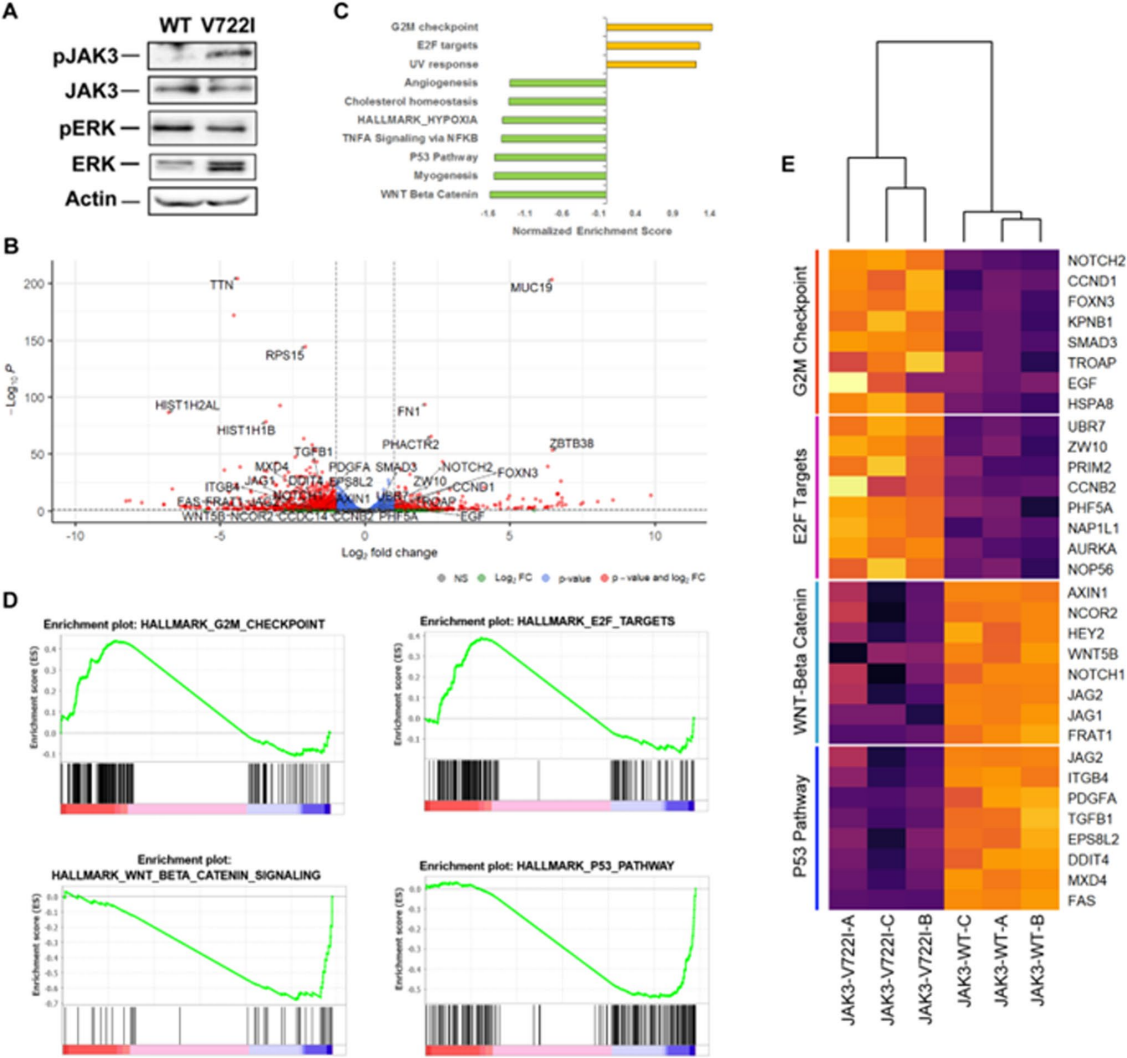
**A** Western blot of representative JAK3 WT and V722I lines showing activation of JAK3 axis. Actin was used as a loading control. **B** Volcano plot representing the differentially expressed genes in JAK3-V722I versus JAK3-WT cell models. Red circles represent genes with |Log2 fold change|> 1 and –Log10 Benjamini-Hochberg (BH)-Adjusted *p* value > 1; blue circles genes with –Log10 BH-Adjusted p value > 1; green circles genes with |Log2 fold change|> 1; grey circles represent genes with |Log2 fold change|≤ 1 and –Log10 BH-Adjusted *p* value ≤ 1. **C** Barplot showing all the significant hallmarks identified in GSEA analysis. Orange bars represent positive enrichment; green ones negative enrichment. **D** GSEA plots reporting the two top positively (top) and negatively (bottom) enriched gene-sets significantly enriched in JAK3-V722I versus JAK3-WT cell models. **E** Heatmap reporting the expression level of the top leading genes of four significantly enriched gene-sets in JAK3-mutated and wild-type lines

JAK3-V722I was found in several tumor types 22, 23 and reported both as somatic as well as germline mutation. Since, the role of JAK3 p.V722I in the context of erythroid differentiation is currently unknown, we generated cell-lines expressing JAK3-p.V722I or JAK3-WT. Western blot analysis of extracts from the two lines using an antibody directed against phosphorylated Tyr980, revealed an increased phosphorylation for the mutated line (Fig. 4A). As Tyr980 is one of the main auto-phosphorylation targets in the activation loop of JAK3, an increased signal suggests the activation of the JAK3 pathway in the mutated line. To characterize the differential transcriptional programs of the two lines we performed a whole-transcriptome sequencing analysis, which revealed a profound change in transcript expression, with 284 up and 949 downregulated genes in JAK3-p.V722I (Fig. 4B). Gene-set enrichment analysis highlighted the presence of positively enriched hallmarks associated with G2M transition and activation of E2F-driven signaling (Fig. 4C–E). E2F genes are retinoblastoma (Rb)-regulated transcription factors playing an important role in terminal erythroid maturation. E2F family members, and in particular E2F-2, are able to form a ternary complex with phosphorylated Rb and the master inducer of erythroid differentiation GATA-1. The net effect of this interaction is to steer the differentiation of myeloid hematopoietic precursors toward the erythroid lineage 24. Hallmarks associated with WNT-Beta-Catenin and P53 pathway were also negatively enriched in JAK3-p.V722I lines. Activation of both pathways was reported to be associated with ineffective erythropoiesis and refractory anemia in the context of myelodysplastic neoplasms 25, 26.

### EPAS1-p.P540L and p.F374Y models

EPAS1-p.P540L variant was originally identified in a single patient from a family with a reported history of congenital erythrocytosis 27. This variant is localized near the C-terminus of the EPAS1 protein, very close to Proline 531, which represents the primary site of prolyl hydroxylation by PHD2. In presence of high O_2_ concentration, PHD2 is able to efficiently hydroxylate target proteins, such as EPAS1, causing their ubiquitination and proteasomal degradation. The very close proximity of P540L to the hydroxylation site suggests that this variant may decrease the affinity of PDH2 to its substrate, causing a reduced hydroxylation efficiency and ultimately leading to EPAS1 stabilization. EPAS1-F374Y variant was reported as a gain-of-function mutation showing a decreased binding to VHL protein and a less efficiently ubiquitination as assessed by simulated structural analyses, co-immunoprecipitation and cycloheximide assays 28.

**Fig. 5:**
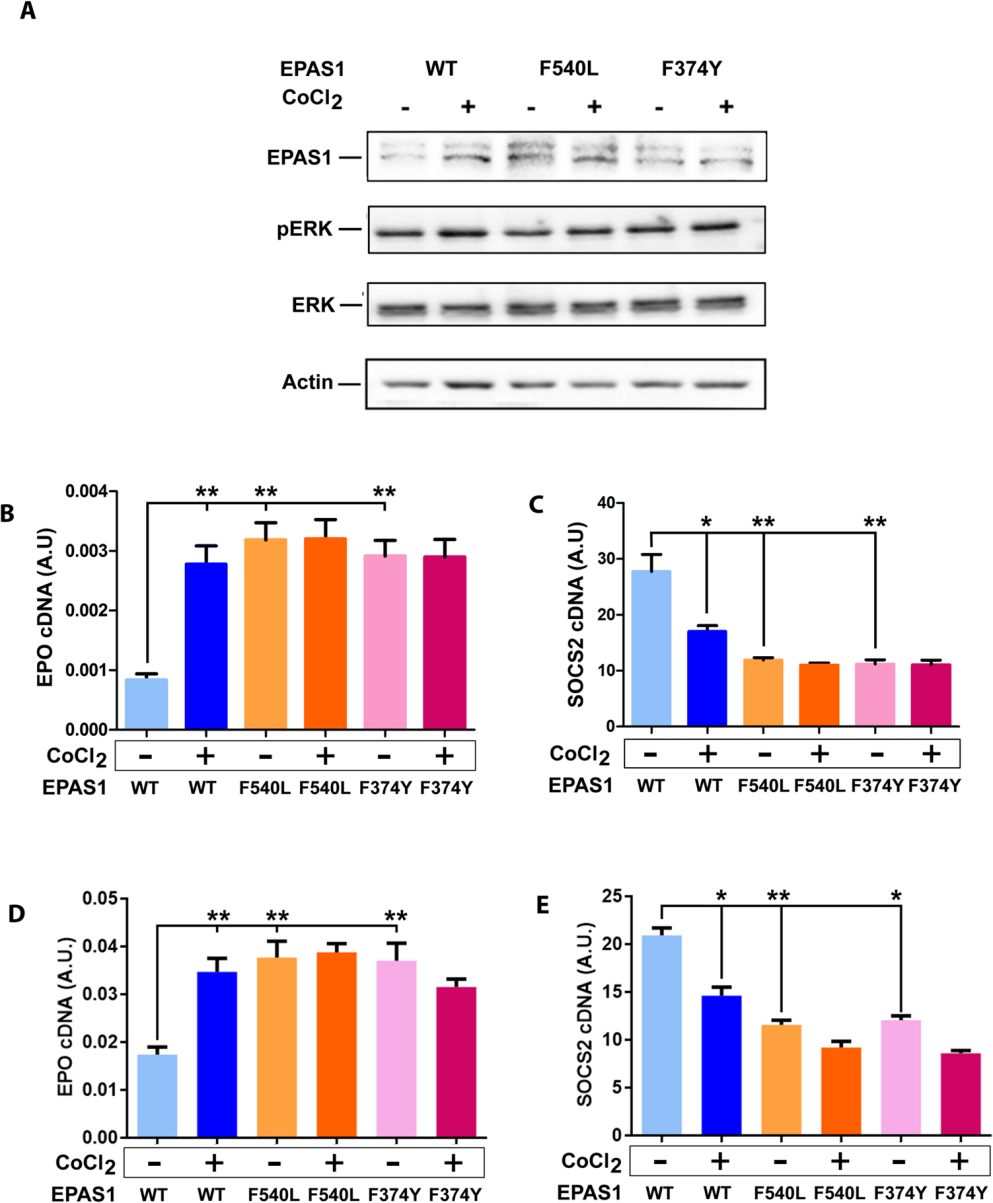
**A** Western blot on lysates of EPAS1-WT, F540L and F374Y cell-lines in presence or absence of CoCl_2_ as a chemical inducer of hypoxia. Actin was used as a loading control. **B** Q-PCR analysis of *EPO* expression in EPAS1-WT, F540L and F374Y cell-lines in presence or absence of CoCl_2_ **C** Q-PCR analysis of *SOCS2* expression in EPAS1-WT, F540L and F374Y cell-lines in presence or absence of CoCl_2_. **D** Q-PCR analysis of Erythropoietin (EPO) expression in HEK293 cell line transiently transfected with EPAS1-WT, F540L or F374Y cell-lines in presence or absence of CoCl_2_ as a chemical inducer of hypoxia **E** Q-PCR analysis of *SOCS2* expression in HEK 293 cell line transiently transfected with EPAS1-WT, F540L or F374Y cell-lines in presence or absence of CoCl_2_. Data were analyzed using two-tailed, unpaired t-tests. *p < 0.05; **p < 0.005; ***p < 0.001. Histograms represent the mean of three replicates; the error bars represent the Standard Error of the Mean (SEM)

To test this hypothesis, we generated stable cell models expressing either EPAS1 wild-type, EPAS1-p.P540L or EPAS1-p.F374Y in the erythroleukemic K562 cell line and we analyzed EPAS1 expression, showing that both P540L and F374Y cause EPAS1 protein upregulation, up to a level comparable to that of a hypoxic condition, simulated by treating cells with CoCl_2_ (Fig. 5A). As EPO is the main transcriptional target of EPAS1, we tested *EPO* expression by Q-PCR, demonstrating an upregulation in both cell lines (Fig. 5B; 3.8-fold for p.P540L, *p* = 0.0015 and 3.5-fold for p.F374Y, *p* = 0.0018). Similarly, we showed that *SOCS2*, a potent suppressor of the JAK2 axis 28 known to be down-modulated by hypoxia 29, was significantly downregulated (Fig. 5C; 2.3-fold for p.P540L, *p* = 0.0064 and 2.5-fold for p.F374Y, *p* = 0.0060).

A similar experiment was performed on the human embryonic kidney cell line (HEK293) transiently transfected with either EPAS1 wild-type, EPAS1-p.P540L or EPAS1-p.F374Y: The same pattern was shown (Fig. 5D: *EPO*: 3.6-fold for P540L, *p* = 0.0017 and 3.1-fold for F374Y, *p* = 0.0016; Fig. 5E: *SOCS2*: 2.8-fold for p.P540L, *p* = 0.0052 and 2.3-fold for p.F374Y, *p* = 0.0063).

### HIF1A-p.P582S model

HIF1A-P582S polymorphism has been already found to be enriched in IE patients; however, its role in HIF1A stabilization has been controversial, with reports suggesting no effect in terms of hydroxylation or protein stabilization 27 and others indicating that HIF1A-p.P582S may lead to an altered transcriptional activity 30, 31.

**Fig. 6:**
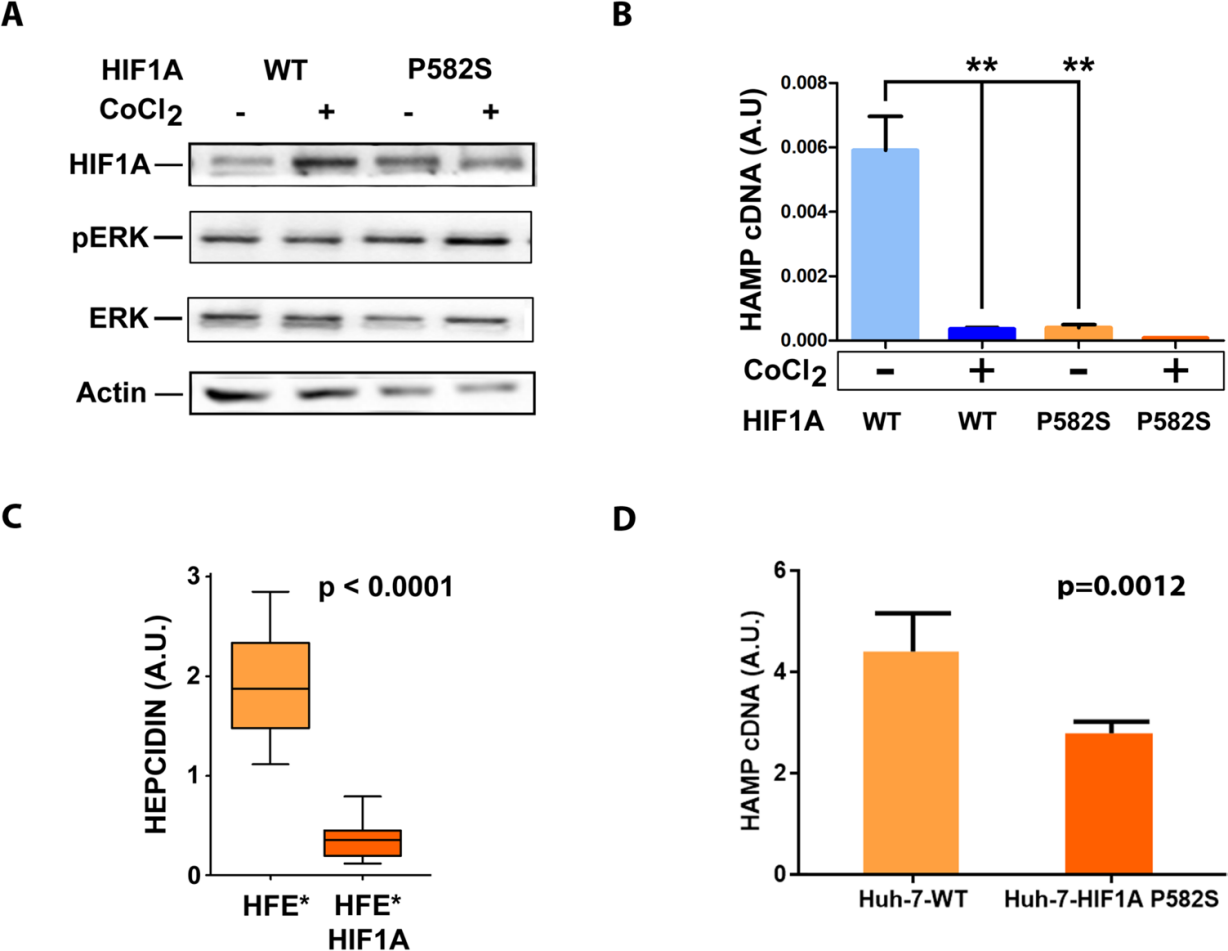
**A** Western blot on lysates of HIF1A-WT and P582S cell-lines in presence or absence of CoCl_2_. Actin was used as a loading control. **B** Q-PCR analysis of *HAMP* expression in HIF1A-WT and P582S cell-lines in presence or absence of CoCl_2_. **C** Analysis of plasma Hepcidin expression by ELISA assay in 3 patients affected by hemochromatosis and homozygous for HFE-C282Y versus 3 homozygous for HFE-C282Y and heterozygous for HIF1A-P582S. The boxplot shows the interquartile range; the whiskers represent the minimum-to-maximum range. **D** Q-PCR analysis of *HAMP* expression in HIF1A-WT and P582S liver HUH7 cell lines (3 replicates). Data were analyzed using two-tailed, unpaired t-tests. *p < 0.05; **p < 0.005; ***p < 0.001. Histograms represent the mean of three replicates; the error bars represent the Standard Error of the Mean (SEM)

To study the specific effect of HIF1A P582S variant, we generated HIF1A-WT and HIF1A-p.P582S cell-models. Interestingly, HIF1A-p.P582S lines showed limited evidence of HIF1A protein stabilization (Fig. 6A). As it has been reported that HIF1A can downregulate liver hepcidin under condition of iron deprivation [32–34, we investigated the effect of HIF1A p.P582S mutation on hepcidin expression by Q-PCR. This analysis revealed a profound suppression of hepcidin expression in P582S as compared to WT (Fig. 6B; 14.7-fold down-modulation; *p* = 0.0065).

To confirm this finding, we analyzed hepcidin levels in the plasma of 6 patients affected by hemochromatosis, 3 of them homozygous for HFE-p.C282Y and 3 homozygous for HFE-p.C282Y and heterozygous for HIF1A-p.P582S. Plasma Hepcidin levels were potently suppressed (5.4-fold down-modulation) in HIF1A-p.P582S cases (Fig. 6C; *p* < 0.0001), in line with our cell-models, hence confirming HIF1A-p.P582S variant as a modifier of HFE-hemochromatosis and possibly explaining why healthy blood donors expressing this variant usually do not show evidence of iron deprivation 33. A similar experiment performed on the human liver cell-line Huh-7 showed a comparable pattern (Fig. 6D; *p* = 0.0012).

**Fig. 7:**
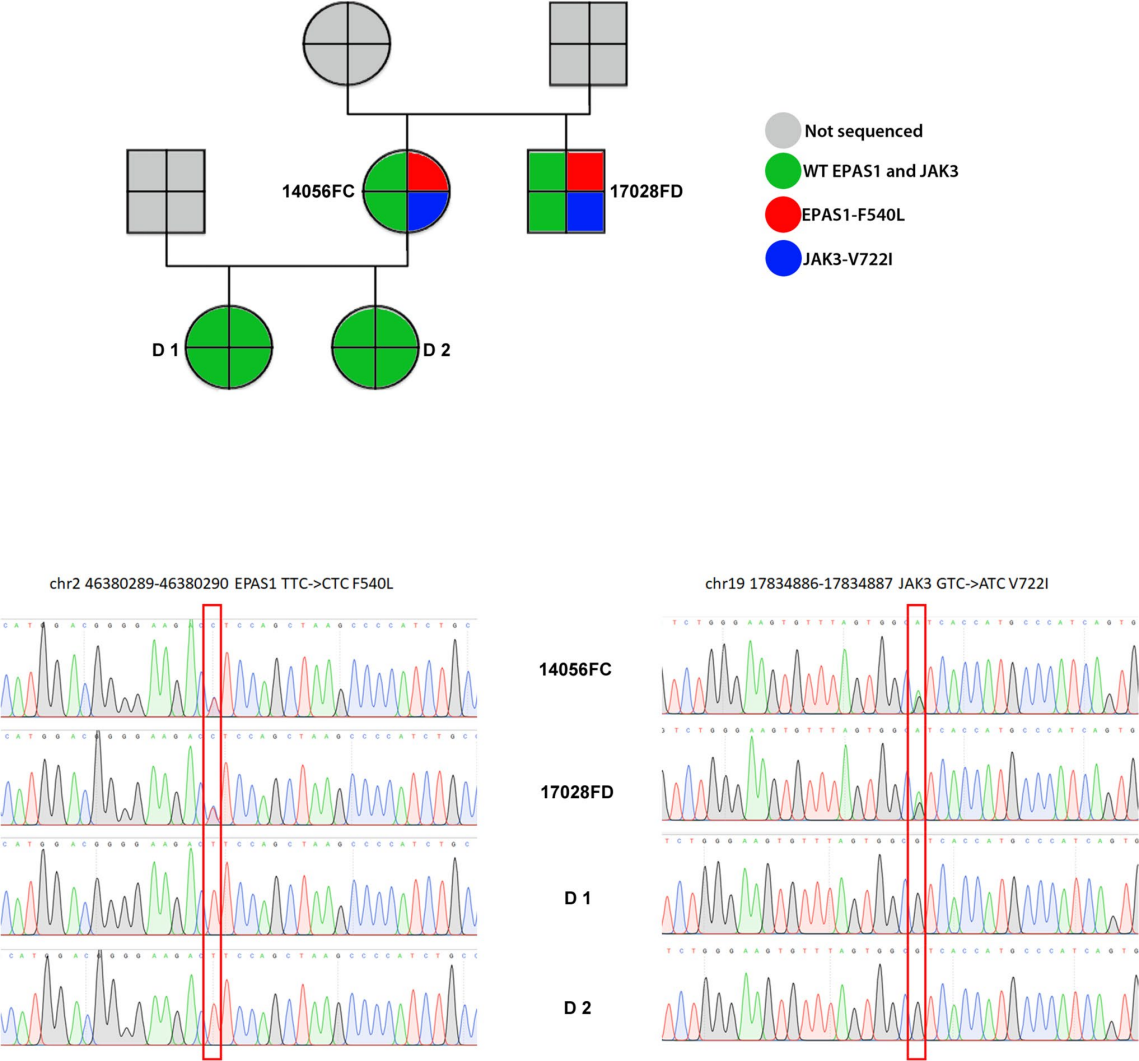
Genetic Pedigree of female proband and his brother, both affected by IE. Exome analysis revealed the presence of JAK3-V722I and EPAS1-F540L heterozygous mutations in both cases. No evidence of the two variants could be found in the two healthy daughters of the proband as shown in Sanger sequencing validation

In the context of our study, we also identified a female patient showing a compound JAK3-p.V722I/EPAS1-p.P540L mutation. Notably, a second family member (brother) was affected by IE, while two daughters of the proband were not affected by the disorder. Genotyping confirmed a complete association between the JAK3-p.V722I/EPAS1-p.P540L genotype and IE (Fig. 7), therefore supporting the hypothesis of a functional, compound effect for both mutations.

## Outcome

After a median follow-up of 7.7 years (range: 0.8–27.4 years), 6 (10.7%) thrombotic events were reported in IE, with an incidence similar to the PV group (*n* = 6, 10.7%, Table 2). This is in agreement with the observation that RBC independently promote arterial thrombosis increasing the rate of platelet deposition and thrombus growth 35 and suggests a more stringent program of phlebotomy (i.e., to maintain Hct < 45%) and a diffuse use of low dose aspirin also in IE patients similarly to PV cases (in our experience, 75% *vs* 98% of IE and PV patients, respectively, received antiplatelet therapy, *p* = 0.0003, Table 2).

Only 2 (3.6%) cases of myelofibrotic evolution were observed in IE patients, none of them occurring in patients carrying somatic variants, with a lower rate than PV group (Table 2). Interestingly, both patients that underwent progression to myelofibrosis shared the presence of the JAK3-V722I variant. Although the cohort is too small to support statistical analyses, the strong activation of the STAT5 pathway exerted by JAK3-V722I, together with the identification of the same variant as a somatic event in several cancers suggests that this mutation, when present as a germline variant, may associate with an increased risk of cancer evolution. Therefore, we hypothesize that germline mutations responsible for the abnormal activation of proliferative pathways may promote the development of yet unknown pro-oncogenic mutations and the ensuing evolution to cancer. Further studies will be required to confirm this hypothesis.

## Conclusions

IE is characterized by an increase in red blood cell mass without an identified cause, with a peculiar clinical phenotype at onset (male, young, isolated erythrocytosis) and an indolent course of disease 7, 8. To date, its diagnosis is based on the exclusion of PV 2, secondary acquired polycythemias and various congenital primary and secondary polycythemias [5–7.

Here we analyzed by paired blood/buccal-DNA exome-sequencing as well as with a NGS myeloid panel a cohort of 56 Caucasian adult IE consecutive patients, to identify germline or somatic mutations responsible for the onset of the disease.

In only 14 (25%) patients, evidence of some somatic variants with low mutation burden (VAF < 10%) was found.

Taken globally our data suggest that a large fraction of IE cases is represented by germline disorders, characterized by the presence of recurrent germline variants occurring on JAK/STAT, Hypoxia and Iron metabolism pathways, among them: JAK3-V722I and HIF1A-P582S. These findings indicate that the presence of specific variants in the pathway mentioned above could be associated with the emergence of an erythrocytic phenotype. We show that JAK3-V722I causes a prominent alteration in the transcriptional program of the target cells, activating pathways responsible for promoting erythroid differentiation and cell proliferation while also protecting hematopoietic precursors by suppressing networks associated with ineffective erythropoiesis. HIF1A-P582S instead causes suppression of hepcidin mRNA synthesis, suggesting a major role for these variants in the onset of IE. The demonstration that a significant fraction of these patients is affected by a genetic disease confirms the need to avoid cytoreductive therapies, unless the presence of a clonal disorder is clearly demonstrated by the presence of somatic variants with high VAF. Also, this finding may pave the way to clinical trials dedicated to the definition of a proper treatment and suggests the opportunity of supporting these patients and their families with dedicated genetic counseling. Finally, despite the absence of a clear evidence of clonality in the majority of IE cases, the incidence of thrombosis is high, comparable to that of PV. This suggests that the main goal of therapy in IE should be focused on reducing the risk of thrombosis by controlling the Hct levels.

## Supplementary Information

Below is the link to the electronic supplementary material.

Supplementary file1 (DOCX 363 kb)

## Data Availability

Raw sequencing data support the findings of this study are openly available in the SRA repository at the following link: https://www.ncbi.nlm.nih.gov/sra/PRJNA965921.
