# Supplementary material for: Idiopathic erythrocytosis: a germline disease?
Source: Clin Exp Med. 2024 Jan 20;24(1):11. doi: 10.1007/s10238-023-01283-y (PMC10799805; doi:10.1007/s10238-023-01283-y)
Supplement: Supplementary file 1 — Supplementary file1 (DOCX 363 kb) [file 10238_2023_1283_MOESM1_ESM.docx]

**Supplementary information:**

**Immunoblot analysis**

Protein extracts (20-40 μg) obtained from cell lysates were separated on SDS-PAGE, transferred to a nitrocellulose membrane, and blotted with the following primary antibodies: JAK3 (5481S Cell signaling; 1:1000), pJAK3 (5031S Cell-signaling; 1:1000), EPAS1 (NB100-122 Novus Biological; 1:500), HIF1a (ab82832 AbCam, 1:1000), ERK (9122 Cell-signaling; 1:2000), pERK (9121 Cell-signaling; 1:1000), Actin (A2066 Sigma-Aldrich, 1:4000). Secondary antibody: anti-mouse anti-rabbit HRP conjugated (Biorad, 1:2000).

**Quantitative Real-Time PCR**

Q-PCR was performed as previously described^1^. The housekeeping gene GUSB was used as an internal reference. EPO (Hs01071097_m1), SOCS2 (Hs00919620_m1), HAMP (Hs00221783_m1) TaqMan® Gene Expression Assays (Thermo Fisher Scientific) were used.

**ELISA assay**

Serum samples were collected and stored at -20°C.

Hepcidin quantification was performed using the ELISA KIT by R&D Systems (DHP250) following manufacturer instructions.

**Statistical analysis**

Continuous variables have been summarized by their median and interquartile range, and categorical variables by count and relative frequency (%) of each category. Comparisons of quantitative variables between groups of patients were carried out by Wilcoxon-Mann-Whitney rank-sum test or Student’s t-test; association between categorical variables (2-way tables) was tested by the Fisher exact test or chi-square test, as appropriate.

**Supplementary Tables:**

**Table 1S:** Primer list used for site direct mutagenesis of analyzed variants.

**Table 2S:** Molecular profile of Idiopatic Erytrocytosis: somatic and germlines mutations

1. **Somatic mutations (VAF < 10%)**

**
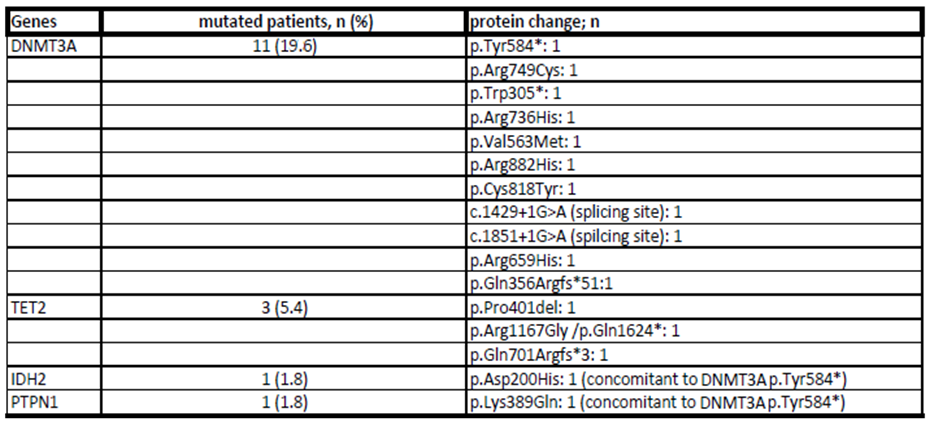
**

1. **Germlines mutations**

**
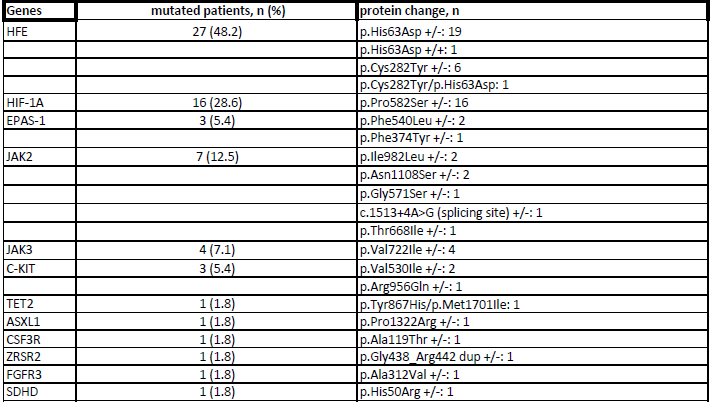
**

**Table S3:** List of candidate pathogenic variants focused on polymorphisms occurring on genes associated with the JAK-STAT pathway, Gene Ontology Accession Term GO:0007259

**Table S4:** List of candidate pathogenic variants focused on polymorphisms occurring on genes associated with the *Response to hypoxia, Gene Ontology Accession GO:0001666*

**Table S5:** List of candidate pathogenic variants focused on polymorphisms occurring on genes associated with the *Cellular iron ion homeostasis Gene Ontology Accession GO:0006879*

**Table S6:** Encode^2^ database for STAT5 occupation by focusing on promoters of genes associated with the *Cellular iron ion homeostasis* ontology.

**

**Database for single patients clinical features and mutations:** a complete database with the clinical and molecular features of our IE cohort. Mutations annotated in the database refers to a specific subset of genes as described in the results.

***References***

1. Piazza R, Valletta S, Winkelmann N, et al. Recurrent SETBP1 mutations in atypical chronic myeloid leukemia. *Nat Genet*. Jan 2013;45(1):18-24. doi:10.1038/ng.2495

2. Birney E, Stamatoyannopoulos JA, Dutta A, et al. Identification and analysis of functional elements in 1% of the human genome by the ENCODE pilot project. *Nature*. Jun 14 2007;447(7146):799-816. doi:10.1038/nature05874
